# Prof. Thomas L. Buckingham

**Published:** 1883-10

**Authors:** 


### OBITUARY.

---

### THOMAS L. BUCKINGHAM, M. D., D. D. S.

**Died—At Philadelphia, Sep. 4th, 1883, of softening of the brain, Thomas L. Buckingham, M. D., D. D. S., in the sixty-seventh year of his age.**

"Thomas L. Buckingham was the oldest of nine children. He was born in New Castle County, Del., on the 9th of March,

1816. At the age of seventeen, after having received an ordinary schooling, he desired to learn the trade of machinist, but his father being a miller, and Thomas the oldest son, desired him to assist in the mill. This he did for a period of three years, at the end of which time his father died. Shortly before his death, however, he had purchased a farm, intending to remove his family thereon, but death intervening, the widow undertook to execute his plans, and Thomas became the head of the family and a farmer.

A few years of farming sufficed for him, when his ambition again changed the channel of his life. Thinking his fortune lay in the direction of commercial business, he entered upon the dry goods trade in Wilmington, Del., with his uncle, but this in a short time proved unprofitable. During all this time he was no doubt undergoing, through his struggles and failures, a preparation for his higher calling; receiving his lessons of patience and perseverance through practical experience, that he might in after life instil into the minds of his many students the same principles he had bought so dearly.

While engaged in the dry goods business he came in contact with Dr. Reynolds, who was practicing dentistry at that time in Wilmington. Then it was that the old fire was rekindled within him, which was started with his desire to learn the machinest's trade; though almost quenched by his father, there was enough yet remaining to urge him to the study of dentistry, in which profession he saw abundant opportunity for the exercise of all his mechanical skill and ingenuity.

Under the tuition of Dr. Reynolds he acquired all the then known art of dentistry, or enough, at least, to warrant him in in starting for himself.

He shortly afterwards opened an office in Wilmington, and in 1846 came to Philadelphia, entering into a partnership with Dr. Lee, though only for one year.

A few years subsequently he was chosen by the State Society as one of the five gentlemen to be recommended to the Board of Trustees for the Professorships in the Philadelphia College of Dental Surgery. This institution was opened in 1852, Dr. Buckingham occupying the chair of Mechanical Dentistry. Four years afterwards, some difficulty arising between the Faculty and the Board of Trustees in relation to the granting of honorary diplomas without the consent of the Faculty, the college was abandoned.

The old Faculty then made application to the Legislature for a charter to establish a new school, and in November, 1856, they entered upon their duties as Faculty of the Pennsylvania College of Dental Surgery, which institution has ever since continued under the same name, and with which Dr. Buckingham has been identified from its incorporation until his death. He was Dean of the Faculty from '57 to '61, and from '65 to '72. He graduated in medicine in 1851 from the Philadelphia College of Medicine, and in 1853 he received his dental diploma from the Baltimore College of Dental Surgery. In 1860 he served as President of the American Dental Convention, and in 1874 as President of the American Dental Association.

Professor Buckingham has been a faithful teacher for thirty-one years, endeavoring to teach to all who came to him those plain truths as he had received them, and instilling into the minds of his students not only a knowledge of dentistry, but those many virtues of a true manhood which in him were as numerous as the gray hairs of his head.

He was particularly popular with his classes, and we doubt if any graduate of the Pennsylvania College has aught but good to say of his dead Professor. His popularity was by no means confined to his students; few members of the dental profession were more widely or favorably known, and in our conventions and professional gatherings his genial face and frank, open expressions of his honest convictions will be sadly missed. In his social life he had a many admirers. He was a man looked up to and respected by all. He was a prominent member of the Masonic fraternity, where he had hosts of friends who mourn his loss."—*Dental Practitioner.*

It is with great regret that we record the death of Dr. Buckingham, whose name as a dental teacher had become so familar throughout the many years of our connection with dental schools.

We heartily endorse the following remarks which close an excellent obituary notice in the October Number of the *Dental Cosmos:*

"He leaves behind him the record of a long and honorable service, and when the roll of those wno have contributed to the rise and progress of modern dentistry is made up, the name of Thomas Lea Buckingham will not be the last."

EDITOR OF JOURNAL.

---

### MONTHLY SUMMARY.

